# Non-invasive imaging of plant roots in different soils using magnetic resonance imaging (MRI)

**DOI:** 10.1186/s13007-017-0252-9

**Published:** 2017-11-17

**Authors:** Daniel Pflugfelder, Ralf Metzner, Dagmar van Dusschoten, Rüdiger Reichel, Siegfried Jahnke, Robert Koller

**Affiliations:** 1 Institute of Bio- and Geosciences, IBG-2: Plant Sciences, Forschungszentrum Jülich GmbH, Wilhelm-Johnen- Str., 52425 Jülich, Germany; 2 Institute of Bio- and Geosciences, IBG-3: Agrosphere, Forschungszentrum Jülich GmbH, Wilhelm-Johnen- Str., 52425 Jülich, Germany

**Keywords:** Root, MRI, 3D imaging, Soil texture, Phenotyping

## Abstract

**Background:**

Root systems are highly plastic and adapt according to their soil environment. Studying the particular influence of soils on root development necessitates the adaptation and evaluation of imaging methods for multiple substrates. Non-invasive 3D root images in soil can be obtained using magnetic resonance imaging (MRI). Not all substrates, however, are suitable for MRI. Using barley as a model plant we investigated the achievable image quality and the suitability for root phenotyping of six commercially available natural soil substrates of commonly occurring soil textures. The results are compared with two artificially composed substrates previously documented for MRI root imaging.

**Results:**

In five out of the eight tested substrates, barley lateral roots with diameters below 300 µm could still be resolved. In two other soils, only the thicker barley seminal roots were detectable. For these two substrates the minimal detectable root diameter was between 400 and 500 µm. Only one soil did not allow imaging of the roots with MRI. In the artificially composed substrates, soil moisture above 70% of the maximal water holding capacity (WHC_max_) impeded root imaging. For the natural soil substrates, soil moisture had no effect on MRI root image quality in the investigated range of 50–80% WHC_max_.

**Conclusions:**

Almost all tested natural soil substrates allowed for root imaging using MRI. Half of these substrates resulted in root images comparable to our current lab standard substrate, allowing root detection down to a diameter of 300 µm. These soils were used as supplied by the vendor and, in particular, removal of ferromagnetic particles was not necessary. With the characterization of different soils, investigations such as trait stability across substrates are now possible using noninvasive MRI.

**Electronic supplementary material:**

The online version of this article (10.1186/s13007-017-0252-9) contains supplementary material, which is available to authorized users.

## Background

Roots play a pivotal role for plant performance. Especially with respect to urgent topics such as global warming and targeted plant breeding, improved nutrient and water use efficiency by the root systems are key for sustainable agriculture and food security. Besides different approaches to phenotype roots, such as aeroponics, hydroponics, paper pouches, and rhizotrons [1–4], visualization of roots in its natural 3D environment in soil is becoming more and more applicable in root research. To visualize the 3D structure of a root system grown under more natural conditions, tomographic methods such as X-ray computer tomography (X-ray CT) [5] or magnetic resonance imaging (MRI) [6], are needed to overcome the opaque nature of the soil. For both 3D imaging techniques, the choice of the substrate has important consequences. Using X-ray CT the segmentation of the roots from the soil is challenging [7]. To ease image processing, artificial substrates [8] or well defined soil moisture [9] are sometimes employed which may be limiting for certain experiments.

For MRI, the substrate has an impact on the image acquisition itself. Here the magnetic properties of some soils suppress the soil water signal [6, 10]. With only root water signal left, the segmentation of the root from the soil is thus realized as a part of the image acquisition. Due to this inherent, physical segmentation even roots well below the image resolution can be identified [6]. However for some soils the magnetic properties such as the presence of ferromagnetic particles deteriorate root imaging with MRI [10]. Soil moisture can also influence the MRI root image quality. At high moisture levels, the suppression of the soil water signal may fail, preventing the analysis of thin roots below image resolution [6]. As the chosen growth substrate has an important role in MR image generation, labs specializing in MRI root imaging thus typically use a single, well studied soil substrate. These substrates resemble natural soils to different degrees, ranging from pure sand [11, 12], over a mixture of sand, peat, and kaolinite clay [13] to a mixture of sand and agricultural soil [6]. The latter one, termed ‘NMR soil’, is the standard substrate in our lab. To investigate the impact of different soils on root development and the stability of root traits across substrates it is necessary to establish multiple soil substrates applicable for MRI root imaging. Before being used in experimental series, each different soil has to be tested and characterized. Although Rogers and Bottomley [10] already identified multiple agricultural soils with excellent imaging capabilities, these soils are not commercially available. The aim of this study is to identify natural soil substrates covering a range of soil characteristics which are suitable for root phenotyping using MRI and are commercially available for the scientific community or breeders. Therefore we tested a set of diverse natural soil substrates with long-term availability offered by the ‘Landwirtschaftliche Untersuchungs- und Forschungsanstalt’ (LUFA) in Speyer [14]. Together with two artificially composed substrates previously used for MRI root imaging [6, 13] we systematically compared the effect of these substrates on MR imaging. We also considered magnetic properties of the substrates, soil moisture, and root diameters since they all may have additional effects on MR imaging. In order to cover this range of parameters, we employed harvested and subsequently buried roots as calibrated phantoms for detection as well as actual plants grown in the substrates under different conditions.

## Materials and methods

### Characteristics of tested soils

**Fig. 1:**
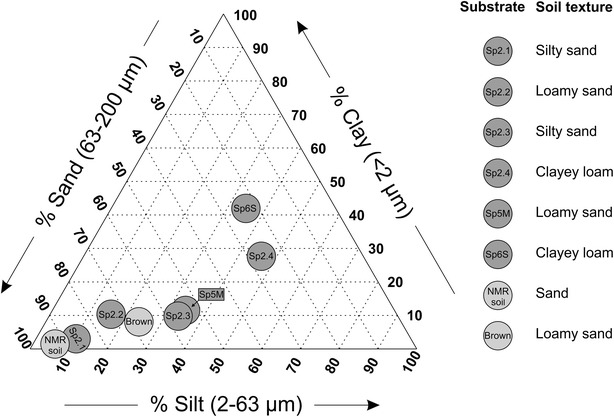
Characterization of used substrates with particle size distribution according to German DIN

In this study we quantified the effect of six different long-term available natural soil substrates offered from the LUFA (Speyer, Germany) [14] on root trait extraction from MRI data. These soil substrates consist of natural soils which were sampled from 0 to 20 cm depth and sieved to 2 mm before shipping. Besides this homogenization, the natural texture and organic composition of the natural soils is preserved. The soils were taken from areas under agricultural use without application of pesticides, biocidal fertilizers, or organic manure for at least 5 years. Mineral fertilizers were used until 3 month before sampling [14]. These ‘standard soils’ span a broad range of soil textures (Fig. 1). Values for organic carbon content, nitrogen content, pH, cation exchange capacity, and particles size distribution are supplied by the LUFA (see Additional file 1). Additionally we employed two artificially composed substrates reported to be suitable for root imaging in MRI: Brown’s soil, a mixture of 50%_vol_ fine sand, 30%_vol_ peat moss, and 20%_vol_ kaolinite clay [13], and our current lab standard NMR soil, a mixture of 33%_vol_ demagnetized agriculture soil and 67%_vol_ coarse sand [6].

Soil moisture was characterized relative to the maximum water holding capacity (WHC_max_) which we defined as the amount of water which is retained by soil against gravity under laboratory conditions. WHC_max_ was determined according to Öhlinger [15]. Therefore air-dry soil was filled into 50 ml centrifuge tubes (BD Biosciences, Bedford, MA, USA) which were cut open at both ends. The tubes with soil were placed on fine sand of 3 cm height. The samples were flooded for 48 h. After a drainage period of 24 h, the remaining water content (WHC_max_) was determined.

Natural soils can contain ferromagnetic particles [10]. To test the effect of these particles on MR image quality, a subsample of each tested soil was demagnetized by removing ferromagnetic particles from the soil. Therefore a magnet (1.42T remanences, size 45 × 45 × 15 mm^3^, from Magnetic Components Engineering, Bedfordshire, UK) was repeatedly moved over a one-grain-thick layer of soil until all particles adhering to the magnet were removed. The soil names and characteristics of the used substrates are summarized in Table 1 and Fig. 1.

**Table 1:** Soil classification and physical soil properties of the used substrates (mean ± standard deviation, N = 4)

Substrate (short name)	NMR soil	Sp2.1	Sp2.2	Sp2.3	Sp2.4	Sp5M	Sp6S	Brown’s soil
Substrate (full name)	N/A	Sp2.12915	Sp2.22915	Sp2.33015	Sp2.42915	Sp5M2915	Sp6S2915	N/A
Soil texture according German DIN	Sand	Silty sand	Loamy sand	Silty sand	Clayey loam	Loamy sand	Clayey loam	Loamy sand
Dry density (g/cm^3^)	1.84 ± 0.05	1.62 ± 0.06	1.25 ± 0.07	1.57 ± 0.05	1.10 ± 0.02	1.36 ± 0.02	1.20 ± 0.08	1.39 ± 0.02
Density at WHC_max_ (g/cm^3^)	1.69 ± 0.04	1.43 ± 0.05	1.13 ± 0.02	1.36 ± 0.05	1.00 ± 0.03	1.21 ± 0.02	1.08 ± 0.07	1.26 ± 0.03
WHC_max_ (%_vol_)	26.3 ± 0.4	31.8 ± 0.7	35.9 ± 0.6	33.5 ± 0.4	36.1 ± 0.6	35.8 ± 0.5	36.1 ± 1.2	32.9 ± 0.4
WHC_max_ (%_mass_)	15.6 ± 0.4	22.3 ± 1.3	31.9 ± 0.8	24.6 ± 0.9	36.2 ± 0.6	29.7 ± 0.6	33.6 ± 1.2	26.1 ± 0.8
Ferromagnetic particles content (%_mass_)	N/A	2.5	2.1	2.5	11.7	5.3	25.3	N/A

The denoted short names are used in the text

### MRI measurement

All MRI measurements were performed on a vertical 4.7T magnet equipped with a Varian console (Varian, Palo Alto, CA, USA). Due to the vertical magnet plant images could be acquired in the natural vertical orientation. A radio-frequency coil with an inner diameter of 100 mm (Varian, Palo Alto, CA, USA) was used. MR images were acquired with our standard protocol consisting of a Spin-Echo Multi-Slice (SEMS) sequence with the following parameters [6]: Repetition time TR = 2850 ms, Bandwidth BW = 156 kHz, horizontal slices with 1.0 mm thickness, in-plane resolution 0.5 × 0.5 mm^2^, matrix size 192 × 192 × 100, Echo time TE = 9 ms, two averages. The measurement time was approx. 20 min for a soil volume of 9.6 × 9.6 × 10 cm^3^. A discussion of the possible implications of the chosen MRI hardware and imaging protocol on the minimal detectable root diameter is provided in the Additional file 1.

### Soil screening

#### Detection of buried roots

To assess how different soils affect imaging of lateral and seminal roots, i.e. typical root classes within a root system, we excavated two barley (*Hordeum vulgare* L. var Barke) root systems 1 month after sowing. Subsequently the roots were scanned and analyzed by WinRhizo software (Regent Instruments, Ottawa, Canada) in order to identify and select lateral roots with diameters between 250 and 350 µm and seminal roots with diameters between 750 and 1000 µm. One seminal and one lateral root piece of 1 cm length each was buried in a soil-filled scintillation vial (20 ml, PerkinElmer, Waltham, MA, USA, soil moisture = 50% WHC_max_). Four vials per substrate were prepared and MRI root images were acquired. The visibility of the seminal and lateral root segments was assessed visually. In this analysis the natural soil substrates were tested twice: as supplied by the LUFA and demagnetized as described above.

#### Effect of water content on root MRI

To screen the influence of substrate moisture on root MRI barley seedlings (*Hordeum vulgare* L. var Barke) were grown in 20 ml scintillation vials (PerkinElmer, Waltham, MA, USA) maintaining soil moisture at 50, 60, 70, and 80% of WHC_max_ of the respective substrate. The natural soil substrates were employed as supplied by the vendor, i.e. ferro-magnetic particles were not removed. The vials were closed with parafilm (Brand, Wertheim, Germany) to prevent water evaporation. Three days after sowing, the seedlings were imaged with MRI.

### Pot experiment

Barley seeds (*Hordeum vulgare* L. var Barke) were pre-germinated on wet paper tissue in a petri dish. After 1.5 days the seedlings were planted into 30 cm long PVC pots with an inner diameter of 81 mm. The natural soil substrates were employed as delivered by the LUFA. In particular ferromagnetic particles were not removed. Soil moisture was set to 60% WHC_max_ and controlled gravimetrically by adding tap water twice a week if necessary. Fertilizer was applied 11 days after sowing (0.5% Hakaphos green stock solution prepared according to manufacturer instructions, Compo, Münster, Germany, 25 ml per plant). Plants were grown in a climate chamber in a 16/8 h light/dark regime, 20 °C during light, 16 °C during darkness, and constant relative humidity of 60%. Lighting was provided by 400 W HPI lamps and 400 W SON-T lamps (Philips, Hamburg, Germany) alternating every 2 h with 5 min overlap giving a PAR light intensity between 350 and 450 µmol m^−2^ s^−1^ at canopy level. Four replicates per substrate were used except for Sp2.3 where only three seeds germinated. Three weeks after germination plant roots were scanned by MRI and subsequently harvested. Excavated roots were washed from adherent soil, scanned, and analyzed by WinRhizo software (Regent Instruments, Ottawa, Canada).

#### MRI data analysis of the pot experiment

The MRI data was analyzed using NMRooting [6]. For this experiment NMRooting was extended to facilitate manual addition and removal of roots from the automatically extracted root system. After manual correction, the obtained root skeleton was used to calculate root length and root diameter as described in [6]. For calculation of the root diameter, the MRI signal intensity needs to be converted to root volume using an empirical calibration factor. In this analysis we employed the calibration factor determined for our lab standard ‘NMR soil’ for all substrates as a first-order approximation.

Since the root MRI signal is dependent on root thickness, very thin roots may fall below the MRI detection limit. Consequently, the total root length obtained from the WinRhizo analysis is typically larger than the total root length obtained from the MRI data. To estimate the minimal root diameter still visible with MRI we utilized the root length and root diameter information obtained from the WinRhizo analysis. Starting from the thickest root, the WinRhizo root length is summed up until the total root length obtained with MRI is reached. The root diameter at this border is used as an estimate for the MRI cutoff diameter.

The cutoff diameters of the various substrates were individually compared to our lab standard ‘NMR soil’ using a Welch’s *t* test. *p* values smaller than 0.01 were considered significant.

## Results

### Soil screening

For a rapid overview on the effect of the different substrates and their content of ferromagnetic particles we first buried pieces of barley seminal and lateral roots of known diameter as phantoms and tested how many pieces could be retrieved of each type in the MR images. All seminal root segments could be recovered in six out of eight investigated substrates regardless of soil demagnetization (see Table 2). From the remaining two substrates, only a single segment was not visible in Sp2.4, while less than half of the buried seminal root segments in Sp6S were detectable in the MR images. The thinner lateral roots were not detected in the MR images of Sp2.4, Sp5M, and Sp6S soils. Demagnetization of the soil did not improve the detectability of the lateral root segments (see Table 2).

**Table 2:** Recovery of root segments buried in different substrates measured with MRI

Substrate	NMR soil	Sp2.1	Sp2.2	Sp2.3	Sp2.4	Sp5M	Sp6S	Brown’s soil
Seminal roots unprocessed soil	4	4	4	4	3	4	2	4
Seminal roots demag. soil	N/A	4	4	4	4	4	1	N/A
Lateral roots unprocessed soil	3	4	4	3	0	0	0	4
Lateral roots demag. soil	N/A	4	4	4	0	0	0	N/A

From an excavated root system lateral roots (diameter 250–350 µm) and seminal roots (diameter 750–1000 µm) were selected. For each class, four 1 cm long root segments were buried in soil and measured with MRI. Listed are the numbers of visually detectable root segments in unprocessed and demagnetized substrates

**Fig. 2:**
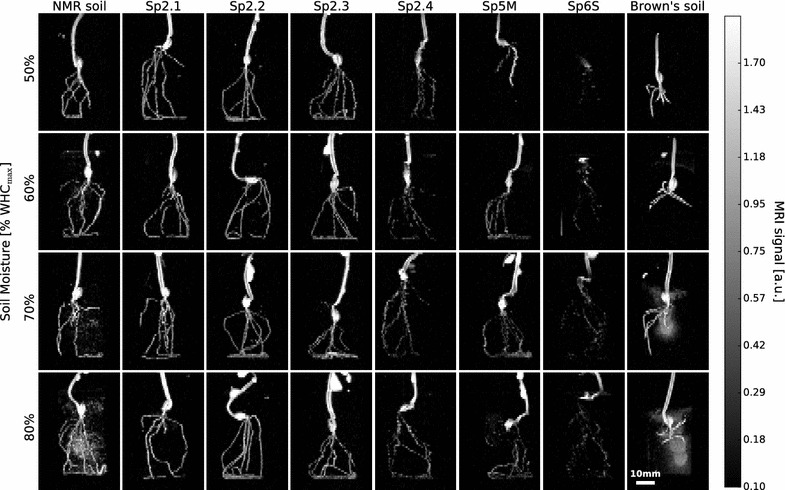
Barley seedlings imaged with MRI 3 days after sowing in eight different substrates at four different soil moisture levels. Image resolution: 0.5 × 0.5 × 1.0 mm^3^, MRI noise level σ = 0.031

For testing the combined influence of soils and soil moisture on MR imaging of real roots we grew barley directly in small scintillation vials and imaged them at four different soil moisture levels (Fig. 2). Three days after sowing seminal roots were visible in all plants as we verified by excavation following the MRI measurement. Seminal roots could be detected in seven out of the eight tested substrates (Fig. 2). For Sp6S a clear delineation of the root system was not possible. For Sp2.4 and Sp5M, the root system could be visualized, although some artifacts, such as missing root parts, are apparent. Furthermore the MRI signal intensity of the roots in these two soils is already close to the noise level, suggesting that thinner roots such as laterals will not be visible in these soils. Soil moisture had a different effect on the eight substrates. For the natural soil substrates image quality did not vary in the investigated soil moisture range of 50–80% WHC_max_. In the artificially composed substrates NMR soil and Brown’s soil, however, soil water became visible at ≥ 70% WHC_max_. Although the roots are still visible this residual soil water signal greatly hinders root segmentation and analysis.

### Pot experiment

For quantitative comparison of the effects of those substrates suitable for MR imaging we grew barley plants for 3 weeks in larger pots and compared roots length and diameter extracted from MR images with WinRhizo data obtained after harvest.

**Fig. 3:**
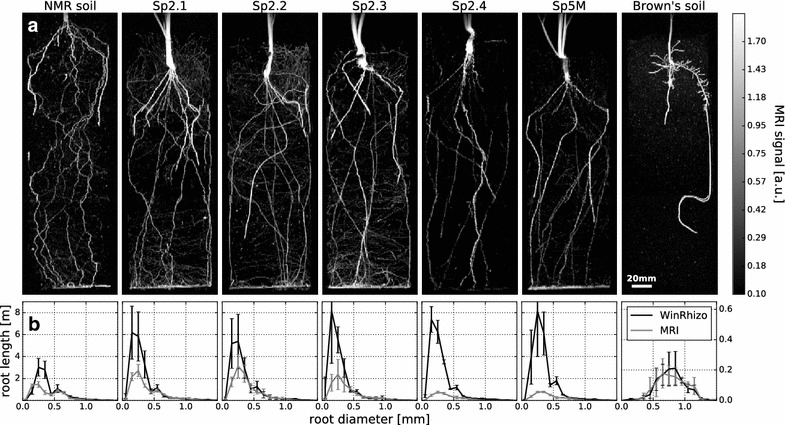
Barley plants were imaged using MRI 3 weeks after sowing. **a** Maximum intensity projections of the 3D image data (Image resolution: 0.5 × 0.5 × 1.0 mm^3^, MRI noise level σ = 0.033). Branching lateral roots are visible in NMR soil, Sp2.1, Sp2.2, Sp2.3, and in Brown’s soil. In Sp2.4 and Sp5M, the soil reduced the MRI signal obtained from the roots, shifting the lateral root signal below the MRI detection limit. **b** Comparison of root length plotted against the root diameter as determined for the whole plant by MRI and WinRhizo after harvest. For better visualization, the y-axis (root length) of Brown’s soil has a different scale than the substrates. In Sp2.4 and Sp5M, the reduced root signal leads to an underestimation of root thickness in the MRI analysis. Error bars: ± 1 SD

**Fig. 4:**
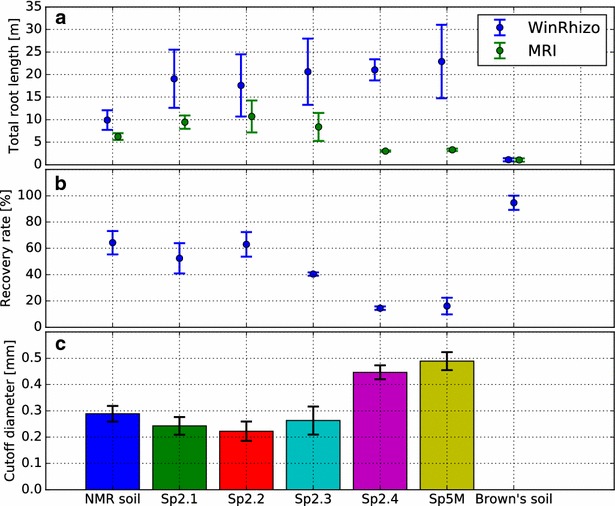
**a** Total root length extracted from Magnetic Resonance Imaging (MRI) data and after excavation and scanning in WinRhizo. **b** Total root length from MRI data relative to excavated root systems. For Sp2.4 and Sp5M, < 20% of the total root length was visualized in MRI. **c** Accordingly, the minimal root diameter detectable in MRI is significantly increased for Sp2.4 and Sp5M substrates compared to our lab standard NMR soil (Welch’s *t* test, *p* < 0.01). For Sp2.1, Sp2.2, and Sp2.3 we found no significant difference to NMR soil although a trend to lower cutoff values is visible. The limit could not be determined for Brown´s soil since here no thin roots were developed by the investigated plants. Error bars: ± 1 SD

Seminal roots could be detected in all tested substrates (Fig. 3a). Branching lateral roots can be visually identified in NMR soil, Sp2.1, Sp2.2, Sp2.3, and in Brown’s soil (Fig. 3a). Comparison with data from WinRhizo, however, shows that in general not all roots were recovered by MRI (Fig. 4a, b). An exception to this is Brown’s soil where the barley plants produced only comparatively thick roots which were almost all detected by MRI (95% recovered root length, see Fig. 4b). Additionally overall root length was shortest (Fig. 4a) and plant development was delayed in comparison to other substrates (data not shown).

For NMR soil, Sp2.1, Sp2.2, and Sp2.3 the minimal root diameter still visible in MRI ranged between 200 and 300 µm (Fig. 4c) which is in line with previous findings [6]. Compared to our lab standard NMR soil there is a trend to lower values in Sp2.1, Sp2.2, and Sp2.3. These differences are, however, not statistically significant (*p* values of 0.090, 0.033, and 0.511, respectively). For Sp2.4 and Sp5M, the minimal detectable root diameters were significantly increased compared to our lab standard NMR soil, ranging between 400 and 500 µm (*p* values 0.00024, 0.00013). In Brown’s soil all roots were visible in the MR images and consequently a cutoff diameter could not be determined.

## Discussion

All substrates tested here enabled root imaging with MRI at a quality that would allow quantification of root traits except for Sp6S. However qualitative and quantitative observations show distinct differences between the effects of the different substrates on MR imaging and to a certain extent also on plant development.

Being able to detect a root in soil depends on the type of substrate as well as on the diameter of the root. Thick roots provide a large MRI signal and are easy to detect. For smaller root diameters the MRI signal may decrease and, at some point, drop below the detection threshold. For this cutoff diameter a value of 200–300 µm has been reported for barley grown in NMR soil [6]. In our experiment the diameters of barley lateral roots were around 300 µm and thus close to the MRI detection limit. Being able to resolve barley lateral roots can thus serve as a simple indicator for MR image quality. Overall, from the eight investigated substrates five (NMR soil, Sp2.1, Sp2.2, Sp2.3, and Brown’s soil) were suitable to resolve lateral roots of barley plants while two (Sp2.4 and Sp5M) could still be used to visualize the thicker seminal roots, which still may represent valuable information about the total root system. Only one substrate (Sp6S) was unsuitable for root MRI. High quality root MR images can be obtained in natural soil substrates without any further processing such as demagnetization of the soil (Fig. 3a). Interestingly, even complete demagnetization of the soil did not improve the detectability of lateral roots (Table 2), suggesting that factors beyond ferromagnetic particles also play a critical role for image quality. On the other hand large ferromagnetic particles would lead to strong image artifacts such as large gaps in the roots. Although we did not encounter any of those particles in our experiment they might still be infrequently present in agricultural soils. Coarse removal of ferromagnetic particles such as suggested in [10] or [6] may therefore be beneficial for image quality in any case. Not all factors influencing root image quality have yet been identified. Soil particle size is probably one of these factors considering that in our study soils with high clay and silt content yielded the worst image quality (Sp6 s and Sp2.4). On the other hand soils with very similar particle size distributions (Fig. 1, Sp5M and Sp2.3) showed contrasting image quality. Magnetic susceptibility or the particle microstructure might also be of relevance for image quality. Since not all factors influencing image quality are known soils have to be tested empirically before being used in an MRI root study.

We did not observe an influence of soil moisture on the acquired MRI root signal in the investigated range (Fig. 2). However an important factor for both image quality and contrast is the suppression of the soil water signal. In the artificially composed substrates NMR soil and Brown’s soil, soil water became visible starting at ≥ 70% WHC_max_, masking the roots and thus impeding root analysis. For the tested natural soil substrates no interfering soil water MRI signal was visible in the tested range from 50 to 80% WHC_max_. Furthermore these soils have high WHC_max_ values, thus permitting higher absolute soil water content as compared to the composed substrates. This broader range of useable soil moistures allows for additional flexibility in experimental designs.

In our experiment soil moisture was set relative to WHC_max_, a value which is known to be dependent on a large number of factors such as bulk soil density or the dimension of the pot used for the measurement [16]. Although values for WHC_max_ values are provided for the standard soils [14] we used our own measured values which can be compared across all investigated substrates.

The modular structure of root systems allows them to be extremely plastic and therefore interact with the heterogeneous nature of soils [17]. Indeed, the barley root system investigated here showed variation in size and structure depending on the tested substrate. For NMR soil, a strong tortuosity of the roots was observed which was not present in the other substrates (see Fig. 3a). The wiggling of the roots in NMR soil, which has been shown to be dependent on soil density, might be partly due to the coarse sand contained in this substrate [18].

Although very clear root images in the screening experiment as well as in larger pots could be obtained using Brown’s soil plant development was severely impaired by the compact structure of this substrate as compared to the other tested substrates. This compact structure led to the development of very thick roots (see Fig. 3b, Brown’s soil). Additionally, the total root length measured after excavation was reduced by up to a factor of 20 as compared to Sp5M (Fig. 4a). It has to be noted that Brown’s soil was developed for MRI root imaging of conifer seedlings which, compared to barley, might be better suited to this type of soil [19]. Except for Brown’s soil, similar root diameters were obtained in all other substrates. Contrastingly, mean total root length after excavation showed variations dependent on the substrate, with more than a factor of two between NMR soil and Sp5M (Fig. 4a). The variability of the root system to the soil environment underlines the necessity to have more than one substrate available for root imaging, for example for testing trait stability across substrates.

To extract root diameters from MR images a calibration factor is needed [6]. As this factor was not available for all substrates we determined the root diameters for all substrates using the calibration factor obtained for our lab standard ‘NMR soil’. For Sp2.4 and Sp5M this led to an underestimation of the root thickness in the MRI analysis as the physical soil properties reduced the observable MRI root signal (see Fig. 3b). For the other substrates shown in Fig. 3b the root diameter determined by WinRhizo and MRI are in good agreement down to diameters close to the cutoff diameter, suggesting that the calibration factor implemented for NMR soil can also be used for these substrates.

As MRI offers a range of different options to generate different image contrasts it has to be noted that here we only investigated the possibilities of root imaging with our standard protocol [6]. For other protocols such as e.g. ultra-short echo time (UTE) imaging or zero echo time imaging [20] it might be possible to visualize roots even in soils found unsuitable in this study. As these sequences would also visualize soil water, the image contrast in turn might become problematic. For root imaging with MRI we had the best results using a T_2_-weighted multi-slice spin-echo sequence combined with a high bandwidth to suppress artifacts resulting from the heterogeneous physical soil properties.

## Conclusions

In almost all investigated substrates 3D root images could be obtained using MRI, albeit with varying image quality. Out of the six tested, long-term available, natural soil substrates, we identified three which can be used in MRI root imaging for root diameters of < 300 µm. Extraction of phenotypic data such as total root length and root diameter has been established in these substrates. This enables investigations such as root trait stability over multiple substrates which are of high relevance to e.g. breeding programs.

## Additional file

**Additional file 1.** Standard soil characteristics and discussion of the effects of the choosen MRI parameters on root images.
