# Supplementary material for: Non-invasive imaging of plant roots in different soils using magnetic resonance imaging (MRI)
Source: Plant Methods. 2017 Nov 17;13:102. doi: 10.1186/s13007-017-0252-9 (PMC5693507; doi:10.1186/s13007-017-0252-9)
Supplement: Supplementary file 1 — Additional file 1. Standard soil characteristics and discussion of the effects of the choosen MRI parameters on root images. [file 13007_2017_252_MOESM1_ESM.docx]

Table 1: Soil characteristics as supplied by LUFA, Speyer. The show values are from May 2015, two months before soil samples were collected for this experiment. Updated values for more recent samples can be found on the LUFA homepage.

| **Substrate** | **Sp2.1** | **Sp2.2** | **Sp2.3** | **Sp2.4** | **Sp5M** | **Sp6s** |
| --- | --- | --- | --- | --- | --- | --- |
| **Organic carbon in % C** | 0.71 ± 0.07 | 1.59 ± 0.13 | 0.67 ± 0.03 | 2.03 ± 0.23 | 1.02 ± 0.08 | 1.77 ± 0.08 |
| **Nitrogen in % N** | 0.06 ± 0.01 | 0.17 ± 0.01 | 0.08 ± 0.01 | 0.22 ± 0.02 | 0.13 ± 0.01 | 0.18 ± 0.01 |
| **pH-value (0.01 M CaCl_2_)** | 4.9 ± 0.3 | 5.4 ± 0.2 | 5.7 ± 0.6 | 7.3 ± 0.1 | 7.3 ± 0.1 | 7.2 ± 0.1 |
| **Cation exchange capacity (meq / 100g)** | 4.2 ± 0.6 | 9.7 ± 0.4 | 7.5 ± 0.9 | 33.0 ± 4.5 | 17.4 ± 3.6 | 26.5 ± 2.6 |
| **Particle size (mm) distribution according to German DIN (%)** | | | | | | |
| **<0.002** | 2.8 ± 0.7 | 8.3 ± 1.3 | 6.6 ± 1.7 | 26.2 ± 1.4 | 11.1 ± 0.7 | 41.7 ± 1.1 |
| **0.002 - 0.006** | 1.6 ± 0.6 | 3.7 ± 0.9 | 5.4 ± 0.8 | 8.0 ± 0.9 | 3.8 ± 1.1 | 8.8 ± 1.3 |
| **0.006 - 0.02** | 3.5 ± 0.3 | 5.2 ± 0.9 | 11.5 ± 0.6 | 14.5 ± 1.2 | 9.5 ± 0.4 | 13.1 ± 0.7 |
| **0.02 - 0.063** | 7.3 ± 0.9 | 8.0 ± 1.3 | 19.0 ± 1.8 | 23.1 ± 0.5 | 22.0 ± 0.8 | 14.3 ± 0.8 |
| **0.063 - 0.2** | 27.5 ± 0.7 | 33.3 ± 2.7 | 24.6 ± 1.5 | 19.3 ± 1.0 | 38.0 ± 1.6 | 9.3 ± 0.6 |
| **0.2 - 0.63** | 55.0 ± 1.3 | 40.9 ± 1.5 | 30.3 ± 0.6 | 7.1 ± 2.1 | 14.4 ± 1.4 | 10.0 ± 1.0 |
| **0.63 - 2.0** | 2.4 ± 0.4 | 0.5 ± 0.1 | 2.7 ± 0.8 | 1.8 ± 0.2 | 1.1 ± 0.2 | 2.8 ± 0.7 |

Effects of the chosen MRI parameters on root images

Detecting roots in soil using MRI poses several challenges. Firstly, thin roots contain only a small amount of water. Thus the overall MRI signal, which is proportional to the total amount of water, is very low and might fall below the detection threshold for very thin roots. Secondly the soil is a mixture of particles, water and air pores, each having very different magnetic susceptibilities [1]. This leads to distortions in the magnetic field, where the distortions are dependent on the susceptibility difference and the geometry of the particles/pores [1]. The distortions of the magnetic field may have different effects, some of which are useful while other are detrimental for root MRI: On length scales below the voxel size magnetic distortions lead to a reduction of the relaxation rates T_2_ and T_2_* of the water near the particles. This reduction in T_2_ causes the suppression of the soil water signal, leading to the excellent image contrast observed in MRI root images [2]. Larger scale distortions, on the other hand, may lead to image artifacts such as distorted roots. Finally very strong magnetic field distortions caused by e.g. larger ferromagnetic particles can lead to a complete signal loss over a considerable volume.

With these challenges in mind, we now discuss the chosen MRI parameters and hardware.

MRI Sequence:

The gradient echo signal decays with T_2_*, which is strongly dependent on magnetic field distortions. Using conventional gradient echo sequences we were not able to detect fine roots in soil due to the short T_2_* values. We therefor employed a spin echo sequence which is more robust against inhomogeneous magnetic fields [2].

Main magnetic field:

Our magnet has a main magnetic field of B_0_=4.7T. The theoretical intrinsic signal to noise ratio scales linearly with B_0_ [3], thus thin roots are potentially better detectable at higher fields. On the other hand magnetic field distortions are also proportional to B_0_ [1] which should be accompanied with increasing image distortions and loss of local signals. For magnetic field strengths other than 4.7T the imaging protocol will have to be adapted. We obtained better root images at 4.7T as compared to our 1.5T machine (data not shown), however to our knowledge the optimal field strength for root MRI is not known.

No shimming was used to obtain the images described here as the field distortions are almost exclusively determined by the soil particles which cannot be corrected for by shimming.

Gradient system:

We used a gradient system with a maximal gradient strength of 300mT/m and a maximal slew rate of 667T/m/s. Using our standard imaging protocol [2] we are far from maximal gradient performance, thus in our case the gradient system does not limit root image quality. For the described protocol we only required a gradient strength of about 50 mT/m and a reduced slew rate of 100T/m/s

Bandwidth:

Increasing the bandwidth on the one hand reduces image artifacts resulting from inhomogeneous magnetic fields. On the other hand, as the signal to noise ratio is proportional to $1/\sqrt{BW}$ [4], this also reduces the signal to noise ratio of the acquired images so thin roots might fall below the detection limit. To reduce image artifacts we employed a high bandwidth (BW=156kHz).

RF coil:

We used a birdcage RF coil with an inner diameter of 100mm to tightly fit our plant pots (outer diameter of 90mm). As the obtainable signal to noise ratio depends on the size of the RF coil, thinner roots might be detectable using smaller RF coils at the cost of smaller pot sizes.

Image resolution:

Although we can detect roots with diameters below the image resolution, a finer image resolution improves the detectability of roots [5]. A smaller voxel size, however, also reduces the signal to noise ratio which is also important for root detection [5]. A finer image resolution could be realized by increasing the signal to noise ratio, e.g. by using a smaller RF coil or by increasing the measurement time. The chosen image resolution of 0.5x0.5x1.0mm^3^ is a compromise taking into account root detectability, useable pot size, and measurement time.

[1] J. F. Schenck, “The role of magnetic susceptibility in magnetic resonance imaging: MRI magnetic compatibility of the first and second kinds,” *Med. Phys.*, vol. 23, no. 6, pp. 815–850, Jun. 1996.

[2] D. van Dusschoten *et al.*, “Quantitative 3D Analysis of Plant Roots growing in Soil using Magnetic Resonance Imaging,” *Plant Physiol.*, p. pp.01388.2015, Jan. 2016.

[3] W. A. Edelstein, G. H. Glover, C. J. Hardy, and R. W. Redington, “The intrinsic signal-to-noise ratio in NMR imaging,” *Magn. Reson. Med.*, vol. 3, no. 4, pp. 604–618, 1986.

[4] E. M. Haacke, Ed., *Magnetic resonance imaging: physical principles and sequence design*. New York: Wiley, 1999.

[5] H. Schulz, J. A. Postma, D. van Dusschoten, H. Scharr, and S. Behnke, “Plant root system analysis from MRI images,” in *Computer Vision, Imaging and Computer Graphics. Theory and Application*, Springer, 2013, pp. 411–425.
